# State-of-the-art gene therapy in epilepsy

**DOI:** 10.1097/WCO.0000000000001349

**Published:** 2025-02-07

**Authors:** Matthew C. Walker

**Affiliations:** Department of Clinical and Experimental Epilepsy, UCL Queen Square Institute of Neurology, University College London, London, UK

**Keywords:** antisense oligonucleotide, focal epilepsy, genetic epilepsies, microRNA, viral vectors

## Abstract

**Purpose of review:**

Gene therapy in epilepsy has undergone a rapid expansion in recent years. This has largely been driven by both advances in our understanding of epilepsy genetics and mechanisms, and also significant advances in gene therapy tools, in particular safe and effective viral vectors. Epilepsy remains an ideal target disease for gene therapy and this review highlights recent developments in this area.

**Recent findings:**

There have been continued advances in the development of antisense oligonucleotide therapies to knock down genes in the treatment of monogenic epilepsies with some now entering clinical trial. However, the greatest recent advances have been in vector gene therapy, which offers a more permanent solution by delivering therapeutic genes directly to the brain as a one-off therapy. In particular, there has been a growth in methods that target focal epilepsy. Such promising approaches close to or in clinical trial include expressing NPY and its Y2 receptor, knocking-down GluK5, a kainate receptor subunit, and the over-expression of Kv1.1, an endogenous potassium channel.

In the future, it is likely that we will take advantage of approaches of regulating more precisely network excitability by using methods such as optogenetics, designer receptors exclusively activated by designer drugs (DREADDs), ‘inhibitory’ glutamate receptors activated by excessive glutamate spill-over, and activity-dependent promoters, which target gene expression to the ‘hyperactive’ neurons.

**Summary:**

Gene therapies offer a novel approach to the treatment of not just genetic epilepsies but any form of epilepsy and may in the future offer an alternative to drug and surgical therapies, allowing more precise, permanent and targeted treatment with fewer adverse effects.

## INTRODUCTION

Gene therapy was originally considered an approach to replace or repair a defective gene [1]. However, in the broader sense, gene therapies are essentially a means of altering the expression of endogenous genes or expressing an exogenous gene, and so can be applied to non-Mendelian disorders and may be particularly suited to neurological disease [2]. Indeed, increasingly, such therapies are not aimed at specific genetic causes but are being used to alter the processes and pathways that can treat the disease. There are multiple ways to achieve this, which I will discuss within this review.

An increasingly popular strategy has been to target messenger RNA (mRNA) transcription. The two main methods to achieve this are the use of antisense oligonucleotides (ASOs) or double-stranded RNA-mediated interference [3]. ASOs are single-stranded deoxyribonucleotides that are complementary to and consequently bind to the target mRNA. ASOs are usually administered intrathecally and can inhibit mRNA transcription, modify mRNA splicing, or promote mRNA degradation. An obvious use is in gain-of-function mutations, such as epilepsy associated with SCN8A mutations, where the resultant overactivity of the sodium channel leads to seizures [4]. ASOs can downregulate the expression of the encoded sodium channel subunit NaV1.6 and have been shown to decrease seizures and mortality in genetic mouse models of SCN8A developmental and epileptic encephalopathy [4]. ASO therapies have also been shown to improve outcomes in mouse models or human stem cell models of other epilepsy-associated syndromes, including progressive myoclonic epilepsies [5], Timothy syndrome [6^▪▪^], and Angelman syndrome [7^▪^]. These ASO therapies can be used prenatally to rescue development [7^▪^] and to direct RNA splicing towards a nonpathogenic variant [6^▪▪^]. More recently, an ASO therapy has been reported in a preprint to have been successfully used in two patients with KCNT1-associated epilepsy of infancy with migrating focal seizures [8].

Paradoxically, the use of ASOs, which increase mRNA degradation, can also be used to increase protein expression in epilepsies associated with loss-of-function mutations. mRNAs are derived from pre-mRNAs which are spliced to produce, sometimes multiple, mRNA variants. Some of these mRNA variants do not encode a functioning protein but still regulate mRNA production. By increasing the degradation of these noncoding mRNA variants, mRNA production and thus protein production increases [9,10]. This has been termed ‘Targeted Augmentation of Nuclear Gene Output’ (TANGO), and has been shown preclinically to prevent SUDEP and reduce seizures in a mouse model of Dravet syndrome in which the therapy increases the expression of the sodium channel subunit, Na_V_1.1 [9]; a trial of such an ASO is underway in people with Dravet syndrome.

An alternative to ASOs is the use of double-stranded RNA-mediated interference, which exploits the cell's endogenous mechanisms for gene expression regulation. This process involves small noncoding RNA sequences, small interfering RNA (siRNA), which binds to the RNA-induced silencing complex (RISC). This complex pairs with the target mRNA and triggers its degradation. siRNA can be delivered directly into the brain or cerebrospinal fluid (CSF) or with cell-penetrating peptides to enable the siRNA to cross the blood-brain barrier. Moreover, such therapies can be used in vector gene therapies (see below).

One of the major limitations of the above therapies is that they need to be given repeatedly, as both the ASOs and siRNAs are degraded over time. An alternative and perhaps more attractive approach is the use of vector gene therapy. The remainder of this article will focus on this approach. 

**Box 1:**
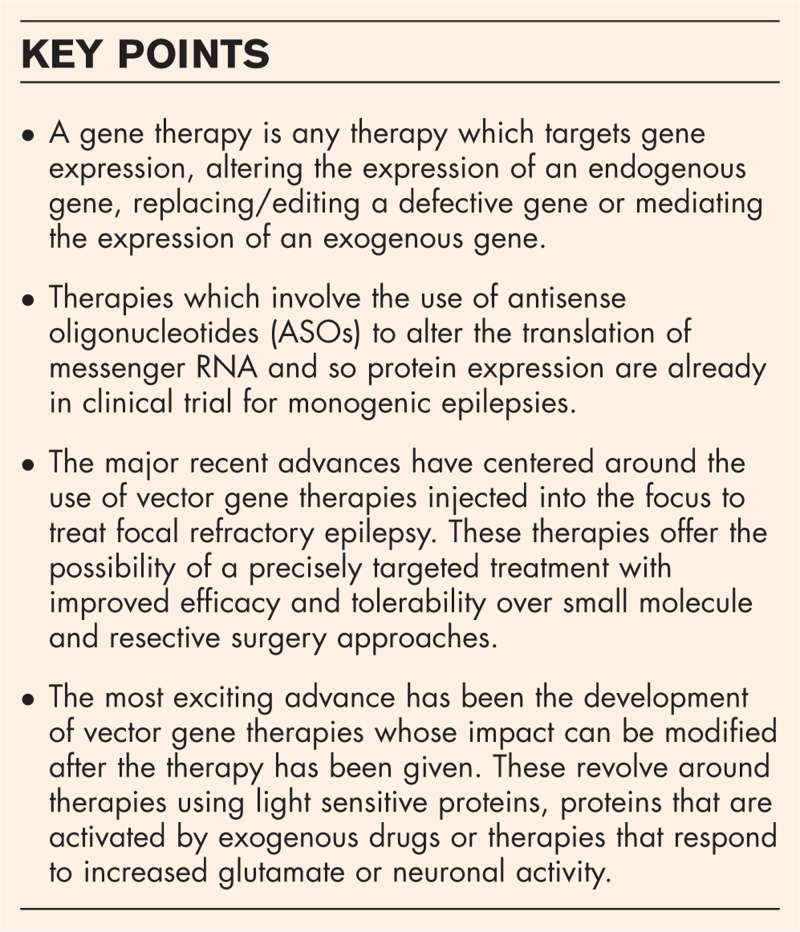
no caption available

## VECTOR GENE THERAPY

Vector gene therapy consists of three key elements. First, there is the vector, which is a replication-incompetent virus. These viruses exhibit tropism for specific cell types, such as neurons, and, even within those cell types, there may be tropism for specific subtypes, such as principal cells or interneurons. Second, there is the promoter, which determines which cell types preferentially express the gene. The third component is the gene of interest. This gene may encode short hairpin RNA (shRNA), which is processed into an siRNA or microRNA, avoiding the need for repeat administration of an siRNA. Alternatively, the gene could encode an endogenous or exogenous protein that regulates cell or network excitability.

Gene therapy in medicine has burgeoned due to advances in safe and effective gene delivery methods [11]. This progress has primarily focused on viral vectors. Viruses have evolved to be efficient carriers of genetic material. Once they enter cells, their DNA is transported to the nucleus, or their RNA is used as a template to synthesize DNA. Viruses consist of a protein capsid, which encloses the viral DNA or RNA. Viral vectors have their genetic instructions for replication removed (i.e. they are replication incompetent) and replaced with the therapeutic RNA or DNA.

Viral vectors differ in their DNA or RNA content and their capsid/envelope structure [12]. Early viral vectors originated from adenoviruses, which can carry large genes, but the adenovirus capsid is highly immunogenic; this has resulted in devastating immune reactions [13]. Current viral vectors are far less immunogenic. In neurological diseases, adeno-associated viruses (AAV), single-stranded DNA viruses, and lentiviruses, RNA viruses with reverse transcriptase, are the main vectors used.

AAV vectors lack a lipid envelope but have low immunogenicity. The capsid binds to a cell surface receptor, enabling entry of the vector into the cell. Different AAV serotypes bind to distinct cell surface proteins, resulting in different tropism for various cell types [14]. Certain AAVs (e.g. AAV1, AAV2, AAV5, and AAV9) are preferentially used to target the central nervous system [14]. AAV-delivered DNA forms episomes, which are not integrated into host DNA [12]. Depending on the serotype, AAVs can spread to varying extents and can even cross the blood–brain barrier [15]. Neutralizing antibodies against AAVs can be triggered by exposure to therapeutic AAVs, and some individuals may have preexisting antibodies. Adeno-associated viruses (AAVs) appear to be the most suitable vectors for the central nervous system (CNS), but they have a limited capacity to carry genetic material (4.5 kb), and at high titers, they have been associated with local inflammation [16].

Lentiviruses can accommodate larger genes (up to 9 kb) and possess a lipid envelope, which, coupled with their larger size, restricts their spread within the brain after injection. This limitation could be advantageous when the target region is close to critical areas for normal brain functions. Most lentiviruses integrate into the host genome, raising the theoretical risk of insertional mutagenesis. However, there are now nonintegrating lentiviruses that achieve stable expression of DNA as episomes, thereby avoiding this potential risk [17].

Due to the restriction in the gene size carried by AAV or lentiviral vectors, there has been continued interest in larger viruses such as adenovirus and herpes simplex virus (HSV) vectors [18], and the creation of completely novel vectors.

The next step is to choose a suitable promoter. This choice influences the cell type in which the gene is expressed and the degrees of expression. While high protein expression may seem desirable, excessive levels of protein production can be cytotoxic. Nonspecific promoters like the CMV (cytomegalovirus) promoter or the synthetic CAG promoter, based on the chicken beta-actin gene promoter with a CMV enhancer, can drive high expression. On the other hand, many cell-specific promoters are comparatively weaker [19]. Common promoters to restrict expression to neurons are the human synapsin (hSyn) promoter and the CaMKII promoter, which further biases expression to excitatory neurons [20]. Restricting expression to inhibitory interneurons can be achieved using the mDlx enhancer [21] or the mGAD65 promoter [22], although these lack specificity for interneuronal subtypes.

## GENE SUPPLEMENTATION AND MODULATION

There are a range of rare monogenic epilepsies which result from loss-of-function mutations. Replacing the defective gene would seem the most straightforward approach. This would need to be achieved using a vector that can spread throughout the brain following intraventricular administration – AAV is ideally suited for this. This approach has been successfully used in a mouse of model of Lafora disease [23^▪^] in which the gene EPM2A can be contained within an AAV9. Injection of the AAV9 into the ventricles led to widespread expression of the target protein and rescued, to some extent, the phenotype [23^▪^]. Similar results have been seen with other monogenic epilepsies such as CDKL5 deficiency [24^▪^]. However, increasing the expression of a target gene can potentially be toxic due to overexpression, especially when the target protein regulates multiple downstream pathways. This is observed with Rett syndrome due to a loss of function mutation of MECP2; gene replacement in mice is toxic [25]. In order to overcome this obstacle, it is necessary to regulate gene expression. This was achieved in MECP2 gene therapy by including within the vector, genes for microRNA that down regulate MECP2 translation – miR-responsive auto-regulatory element (miRARE) [25]. This has proved to be successful in preclinical models of Rett syndrome and is now under clinical trial as an intrathecal, one-time treatment [25,26].

However, the commonest monogenic epilepsy is Dravet syndrome and a significant challenge in Dravet syndrome (and other monogenic epilepsies) is the size of the gene, which, with a suitable promoter, would be too large for an AAV. However, delivering SCN1A using an adenovirus vector is possible and reduces seizure frequency and mortality in a Dravet syndrome mouse model [27]. The adenovirus vector is, however, not suitable for human translation due to its immunogenicity and restricted spread. Thus, different approaches to increase SCN1A in interneurons have been developed. One approach is to split the gene between two AAVs, so that two ‘halves’ of the protein are synthesized with a linker that then joins them together, making use of a process known as intein-mediated protein ligation [28]. In a preprint, this approach has been shown to successfully increase Na_V_1.1 expression and to reduce mortality and epileptiform activity [28].

Gene editing offers an alternative approach. One of the most used gene editing technologies is the CRISPR-Cas9 system. This system utilizes a guide RNA that directs the enzyme Cas9 to a designated location within the genome, where it cuts the double-stranded DNA. As this approach becomes increasingly sophisticated, it can repair or knockout genes. Such technology can be used to specifically inactivate a pathogenic allele. This approach has been shown to be effective in knocking down aberrant SCN8A expression in a mouse model of SCN8A related developmental and epileptic encephalopathy and to rescue the phenotype [29^▪^]. However, the clinical translation of the CRISPR-Cas9 system has been hindered by varying efficacy, and off-target effects. From a translational perspective, the dCas9 system may have greater potential. In this system, Cas9 is mutated to a deactivated or ‘dead’ Cas9, which no longer cuts DNA. Instead, it is fused with gene transcription regulators that either activate or repress genes neighboring the guide RNA binding site. By using a SCN1A-dCas9 activation system in an adeno-associated viral vector, researchers were able to rescue interneuronal excitability, behavior, and attenuate hyperthermic seizures [30]. Despite, the potential of the CRISPR-Cas9 system, a significant drawback is that these therapies relied upon a dual vector approach, as Cas9 or dCas9 and the guide RNA constitute too large a cargo for one AAV.

Lastly, there is a similar approach using an engineered transcription factor designed to upregulate SCN1A gene expression. This was combined with a selective regulatory element (promoter) which restricted expression to GABAergic interneurons. This was effective in animal models and has been shown to be safe in nonhuman primates [31]. This therapy is now entering clinical trials.

In the monogenic epilepsies, targeting the abnormal gene or gene products may not always be an effective strategy. Gene mutations often have a developmental impact, and therefore, reversing the genetic cause postnatally may not be sufficient to undo the effects of that gene mutation on neurodevelopment, indicating that early treatment is likely to be more successful. As a result, even though it may be possible to successfully treat the seizures, there may be no effect on associated comorbidities (e.g. cognitive and behavioral outcomes).

## AN ‘AGNOSTIC’ APPROACH TO GENE THERAPY

As detailed above, there are complex considerations when targeting treatment to a defective gene. These can include incomplete targeting, as only some neurons will be transfected and probably in limited areas of the brain. There are difficulties in controlling expression, with over- and under-expression of the target protein resulting in toxicity or inefficacy, respectively. Moreover, the abnormal gene expression may be involved in the generation of downstream effects, including alterations of the expression of other genes and brain development.

A potentially more tractable problem is focal refractory epilepsy [32]. In this instance, only a limited area of the cortex needs to be treated, and so this therapy lends itself to targeted intraparenchymal injections. These may be directed to one or multiple sites and could include subcortical structures. Moreover, epilepsy is a nonlinear system in which reduction of the excitability of a limited number of neurons may be sufficient to prevent seizures. Since the aim is a reduction of excitability, then strategies can be developed in which neuronal or network excitability is reduced, independent of the underlying cause of the epilepsy (an ‘agnostic’ approach to treatment), which greatly increases the potential population that could be treated. In addition, because such gene therapy treatments modify network excitability without the destruction of tissue (which occurs with resective surgery), they offer a potential therapy that is better tolerated than resective surgery with far fewer cognitive and neurological consequences.

Such a gene therapy approach has been successfully used to overexpress peptides such as galanin, dynorphin, and somatostatin [33–35]. However, most work on over-expressing peptides has been directed towards overexpressing NPY. A challenge is that NPY can have both excitatory and inhibitory effects depending on the receptor on which it acts [36]. Efforts have therefore been directed to overexpress both the peptide, NPY, and its inhibitory receptor, Y2. This strategy has been shown to reduce seizures in models of mesial temporal lobe epilepsy, either when the genes encoding NPY and Y2 are packaged in separate AAVs or within the same AAV [37,38]. This therapy is close to coming to clinical trials.

Decreasing glutamatergic transmission has been shown to be an effective approach in the treatment of epilepsy through, for example, the antiseizure medication perampanel [39]. One of the challenges of antiseizure medications is that they target interneurons and principal cells alike (and so can have pro-seizure effects) and they target the whole brain, leading to adverse effects. Gene therapy, in contrast, can target the excitation of principal cells solely within the seizure focus with potentially improved efficacy and therapeutic specificity. Targeting excitatory transmission with gene therapy has recently been accomplished with downregulation of the kainate receptor subunit, GluK2, through expressing a specific noncoding microRNA [40^▪^]. This has been shown to be effective in an animal model of mesial temporal lobe epilepsy in which there is increased GluK2 subunit expression postsynaptically [40^▪^]. This therapy has been refined so that the vector contains two microRNAs which result in a selective and sustained reduction of GluK2 expression in the hippocampus; this was effective and well tolerated in primates [41]. The clinical trial of this therapy in people with mesial temporal lobe epilepsy is underway.

An even more general approach, which does not rely on region-specific expression of receptors, is to reduce principal cell excitability by overexpressing a potassium channel, Kv1.1 [20,42]. This potassium channel does not ‘turn off’ neurons but modifies their excitability. This modification does not seem to impact function in rodents but has a profound effect on seizures. Moreover, this therapy seems to be equally effective in the neocortex as well as the hippocampus and more recently has been shown to be effective in focal cortical dysplasia due to mTOR hyperactivity (mimicking the human disease) [43^▪▪^].

## ADAPTABLE GENE THERAPY

The problem with most of the therapies above remains the difficulty in regulating the therapy. Insufficient expression may be ineffective, and overexpression may have adverse effects. Strategies that include a mechanism of regulating the activity or expression of a gene therapy are therefore attractive, especially if the therapy is being used in eloquent cortex. One of the earliest such approaches in epilepsy was the use of optogenetics, where the protein expressed is either a channel or pump that is activated by a certain wavelength of light, and the degree of activation depends upon the amount of light detected [42,44]. These channels and pumps can thus be used in a closed-loop fashion, so that when a seizure is detected, light via a fiber optic probe activates, the chloride pump, halorhodopsin, which hyperpolarizes and decreases the activity of principal neurons [45]. Although attractive, this therapy has significant drawbacks for translation. First, it involves not only getting the gene therapy into the focus but also having a light source present there. Second, these light-activated pumps and channels are foreign proteins, derived from algae, bacteria, and archaea, and so there is a concern in the long term of immunogenicity.

A more attractive approach is the modification of the gene encoding the muscarinic acetylcholine receptor, so that the protein expressed is very similar to the endogenous receptor but instead of acetylcholine, it binds and is activated by an exogenous drug. These are termed DREADDs (designer receptors exclusively activated by designer drugs) [46]. DREADDs include the inhibitory hM4D receptor and the excitatory hM3Dq receptor. Most work in epilepsy has focused on expressing the hM4D inhibitory DREADD in excitatory neurons, and this has shown efficacy in neocortical and hippocampal rodent epilepsy models and more recently in a nonhuman primate seizure model [47–49]. This provides a method of getting a specific drug to inhibit only those excitatory cells expressing the DREADD, within the epileptic focus. This permits titration of the therapy by increasing the dose of the drug. Ideally, such a drug would be biologically inert, but, at present, no such drug has been identified. The best candidate drug seems to be olanzapine at a very low dose (avoiding off target side-effects), but this is not ideal [50].

An alternative approach is to use endogenous markers of increased excitability to activate the therapy, as a form of self-regulating closed-loop treatment. Invertebrate glutamate receptors are permeable to chloride and thus, when expressed in human cells, are inhibitory. Such invertebrate receptors lack the machinery to direct their expression into synapses. Thus, expressing such receptors in principal neurons results in the expression of inhibitory glutamate receptors that are extrasynaptic [51]. During physiological synaptic activity, when there is minimal spillover of glutamate beyond the synapse, these receptors are not activated; however, during periods of excessive activity, such as seizures, glutamate spills out of the synapse and activates these extrasynaptic receptors, so inhibiting the seizure activity. Such a therapy has been shown to be effective in focal epilepsy [51], but again, its translation is hindered by the use of an exogenous, potentially immunogenic, protein. Lastly, a significant advance has been the use of an activity-dependent promoter, the c-Fos promoter [52]. This promoter is only activated during increased neuronal activity, such as occurs during seizures, and will only be activated in those neurons involved in the generation of the seizure. This will therefore limit expression of the therapeutic gene to the hyperexcitable neurons involved in seizure generation. This therapy has been tried in rodents using the KCNA1 gene (encoding Kv1.1). Although initially attractive, this therapy is dependent upon hyperexcitability, and so there is the paradox that if you treat the hyperexcitability, you will decrease the efficacy of your therapy. The overexpressed Kv1.1 will eventually be degraded, and the seizures would then be predicted to return. However, in this study, such a therapy was very effective in a rodent model of focal epilepsy [52], and it is, thus, likely that the interictal activity is sufficient to drive overexpression of Kv1.1 to prevent the occurrence of seizures.

## CONCLUSION

Gene therapy approaches for the treatment of epilepsy have rapidly advanced in the last decade with some entering clinical trials. These have largely centered on the use of ASOs in monogenic epilepsies, which require repeat treatments. However, more recently there has been a growth in vector gene therapies, not only to treat monogenic epilepsies, but more importantly to treat all epilepsies. There has been a particular emphasis on focal refractory epilepsy, which remains a large unmet need. The targeting of therapies not just to the focus but also to solely the ‘hyperexcitable’ neurons offers therapeutic precision, which will hopefully permit improved treatment efficacy with far fewer adverse effects, especially compared to our present use of small molecules and resective surgery.

## Acknowledgements

*None.*

### Financial support and sponsorship

*This work was supported by Epilepsy Research UK, Medical Research Council program grants (MR/L01095X/1 r& MR/V034758/) and the National Institute for Health and Care Research University College London Hospitals Biomedical Research Centre.*

### Conflicts of interest

*M.C.W. is a founder shareholder of EpilepsyGtx and is an inventor on patents related to gene therapy in epilepsy. He has undertaken consultancy for: EpilepsyGtx, Seer, UCB pharma and Eisai.*

## REFERENCES AND RECOMMENDED READING

Papers of particular interest, published within the annual period of review, have been highlighted as:

▪ of special interest
▪▪ of outstanding interest
